# Perceptions and quality attributes of goat meat in north-eastern region of Colombia: insights from consumers and farmers

**DOI:** 10.1007/s11250-025-04671-6

**Published:** 2025-09-29

**Authors:** W. S. Sepúlveda, L. X. Estévez-Moreno, D. M. Martínez, N. O. Mancilla, A. Abreu, G. C. Miranda-de la Lama

**Affiliations:** 1 Faculty of Economic, Administrative and Accounting Sciences, Universidad Libre, Socorro, Santander Colombia; 2 Eastern Centre for Productivity and Competitiveness, Bucaramanga, Santander Colombia; 3 Department of Agricultural Sciences and Natural Environment, University of Zaragoza, Zaragoza, Spain; 4 Faculty of Engineering and Agricultural Sciences, Universidad Libre, Socorro, Santander Colombia; 5 Department of Animal Production & Food Science, Agri-Food Institute of Aragon IA2, University of Zaragoza, Zaragoza, Spain

**Keywords:** Consumers, Farmers, Goat meat, Sheep meat, Choice-test, Perceptions

## Abstract

The present study set out to explore the perspectives of consumers and producers on goat meat in Santander, in north-eastern Colombia. The region’s cultural and historical context is characterized by a long-standing tradition of goat production and consumption. A choice experiment involving 85 consumers, complemented by in-depth interviews with 14 producers, revealed a marked preference for goat meat over sheep meat, along with a strong attachment to locally produced meat from Santander. It has been posited by the producers that the distinctive sensory qualities of the product, including its unique taste, are attributable to the extensive grazing practices that take place in the biodiverse *Chicamocha* Canyon. It was determined that goat farming constitutes an ancestral practice that exerts a significant influence on the culinary heritage of the local populace. The alignment between consumer preferences and producer perceptions demonstrates the potential for the development of a Protected Geographical Indication (PGI) for Santander goat meat. The implementation of such an initiative has the potential to safeguard traditional practices, strengthen rural livelihoods, and add market value by differentiating goat meat from other regional products, including sheep meat.

## Introduction

Small ruminant farming is considered a fundamental pillar of rural economies around the world, providing a diverse range of products, including meat, hair, wool, milk, skins and manure (Estévez-Moreno et al. 2019). These farms play an important role in the livelihoods of these communities, being the foundation of small-scale family farming established in areas with underserved or low-income populations (Acero-Plazas 2014). Even though goat meat is not a widely consumed or preferred food in many Western countries, it represents a valuable source of food and economic income for those living in arid and semi-arid areas (de Araújo et al. 2022; Semuguruka et al. 2020). A notable benefit of goat production is its capacity to thrive in terrains that pose challenges to other livestock species, while efficiently converting low-quality pasture resources into high-value food products such as meat (Hamad et al. 2024; Ndona et al. 2024). Consequently, enhanced market access provides income opportunities for smallholder goat producers in arid, semi-arid and mountainous regions (Otieno 2020). Despite the socio-economic role of goat production, it is consumers who, through their purchasing decisions, influence the survival of producers as meat suppliers (Mancilla and Sepúlveda 2017). The integration of these traditional products into the market has been identified as a strategy that enables goat producers to ensure their economic survival and generate income to sustain their livelihoods (Köhler-Rollefson and Mundy 2010). In the context of efforts to establish links between producers and the market, while simultaneously underscoring their provenance, socio-cultural significance, and quality to consumers, a strategic approach is adopted that aims to enhance the competitiveness of these producers when compared with intensive and industrialized systems (Alanís et al. 2022).

In Colombia, goat production is predominantly characterized by extensive or semi-intensive systems, in which small-scale producers specializing in indigenous breeds possess limited access to technological resources. The national inventory comprises 1,136,839 animals. A significant proportion of the national goat inventory, specifically 93%, is concentrated in economically disadvantaged regions in the north-eastern part of the country. This concentration is particularly pronounced in the departments of La Guajira, Magdalena, Cesar (Caribbean region), Santander and Cundinamarca (Andean region) (Rúa et al. 2022). In such circumstances, the annual slaughter of animals in certified slaughterhouses is estimated to exceed 85,000, equivalent to 7,000 tons of meat. However, it should be noted that informal slaughter practices, such as those conducted in butcher shops and private residences, are prevalent in the region. These informal practices have been estimated to potentially double the number of animals declared formally (Acero-Plazas 2014). Colombia’s international trade in small ruminant meat is modest, with the Netherlands Antilles being the sole trading partner to which 60 tons are exported annually. The imports are predominantly sourced from Chile, with smaller quantities also being sourced from Argentina, Uruguay, and the Netherlands. However, the combined imports from these countries do not exceed 15 tons per year. These imports are typically intended for utilization by the nation’s restaurant and retail chains. The estimated per capita consumption of sheep and/or goat meat in the country is approximately 500 grams (Hidalgo 2020). However, at a regional level, consumption is particularly prevalent in the center and north-east of the country, with the indigenous *Wayuu* ethnic group and peasant populations consuming it as a source of subsistence, as well as the rural migrant population in urban centers (Forero et al. 2023). Furthermore, there is an increasing demand for goat meat in the restaurant sector on a national level, as part of the demand for regional identity dishes such as ‘*Cabrito de Santander*’ and *‘Chivo de la Guajira*’ or ‘*Friche*’ (Pinzón et al. 2016). However, it is common practice for these dishes to be prepared with sheep meat (wool or hair breeds) without informing the consumer (Castellón and Fontecha 2018).

In contrast to the extensive scientific literature on sheep meat, there is limited research on consumer preferences regarding the specific geographical origin of goat meat. Sensory evaluations indicate that goat meat is acceptably palatable and desirable, although it tends to be less tender and less juicy than sheep meat due to factors such as pre-slaughter handling and its high ultimate pH (Webb et al. 2005). Additionally, goat meat has species-specific flavor and aroma, as well as a slightly different but acceptable color to consumers (Teixeira et al. 2020). Socio-cultural factors, together with these physical and sensory attributes, influence the preference and acceptance of goat meat, which often occupies specific consumer niches compared to other meats (Estévez-Moreno and Miranda-de la Lama 2022). Within the European Union, Protected Geographical Indications (PGI) have been developed for fresh meat, constituting a quality scheme that identifies a food product originating from a specific place, region or country, and possessing a particular quality, reputation or other characteristics essentially attributable to its geographical origin, with at least one of its production, processing or preparation stages taking place within the defined geographical area (Zjalic et al. 2011). In the Latin American context, this mechanism has proven to be a source of interest, with several local legislative bodies contemplating the implementation of analogous schemes. Nevertheless, despite the economic and commercial benefits of PGIs for livestock producers, some constraints to their effective development have been identified (Cei et al. 2018). Among the most notable are a poor link with the market and the establishment of standards that exceed the technical capacity of producers (Cardoso et al. 2022). Consequently, the correlation between the differentiating effect that geographical origin exerts on consumers and the capacity and potential of producers to manage a PGI has the potential to enhance the probability of success of PGIs (Rabadán et al. 2021). The aim of this study, conducted in northeastern Colombia, is to analyze and explore consumers’ perceptions of goat meat differentiation and its geographical origin, as well as producers’ perceptions of the diversification of their production and its potential to manage a PGI as a tool for differentiation.

## Materials and methods

This article presents the findings of two exploratory studies conducted in the Department of Santander, located in the north-eastern region of Colombia (South America) during the first half of 2021. The first study was conducted in Bucaramanga (7°07′07″N 73°06′58″W), the capital of Santander. This city, with a population of 1,224,257, is the fifth most populated metropolitan area in Colombia (DANE 2018). It is a popular location for food market studies due to its socio-demographic profile, which is considered representative of the Colombian Population Census (Sepúlveda et al. 2016). The second study involved goat farmers in the *Chicamocha* Canyon (6°48′58″N 73°00′36″W), which is located only 54 km S-SE of the city of Bucaramanga. The area in question is approximately 2866.63 km² and is situated at the confluence of 14 municipalities. The climatic conditions of the area are arid, with the *Chicamocha* river acting as a natural border. The dominant vegetation in the *Chicamocha* Canyon is xerophytic scrub, adapted to dry and warm conditions, featuring species such as tuna (*Opuntia* spp.), yarumo (*Cecropia* spp.), cardón (*Stenocereus griseus*), and the spiny tree *Prosopis juliflora*. Xerophytic herbs also appear during the rainy season. In wetter areas near water bodies, tropical dry forests with species like cedar (*Cedrela odorata*) and guaiacum (*Guaiacum officinale*) can be found (Albesiano 2006). The average annual temperature is 25–28 °C, while the average annual precipitation is 731 mm (Rios et al. 2020).

### Study 1: consumers

A choice experiment was conducted with consumers selected through convenience sampling based on their purchase of fresh goat and sheep meat. Initially, a total of 180 consumers were considered, of whom 85 qualified to participate by confirming that they purchased fresh goat or sheep meat. Other studies employing choice experiments with consumers have similarly relied on convenience sampling, a common practice in this type of research (Rossi et al. 2024). The socio-demographic characteristics of the participants in the choice experiment are presented in Table 1. All participants were of an age at which they were legally permitted to make food purchases for their households. The participants were approached in restaurants or markets where goat products are available in the city of Bucaramanga.

**Table 1:** Socio-demographic characteristics of the participants in the study 1

	Percentage
*Gender*	
Male	57.8%
Female	42.2%
*Age range*	
Under 20 years old	9.8%
Between 21 and 30 years old	43.1%
Between 31 and 45 years old	29.4%
Between 46 and 60 years old	13.7%
Over 60 years old	3.9%
*Study level*	
Basic/ primary	5.9%
Secondary/ technical	29.4%
Technologist	31.4%
University	33.3%
*Minimum monthly wage (MMW)*	
Equal to or less than 1 MMW	25.6%
Between 1 (more than 1) and 2 MMW	38.9%
Between 2 (more than 2) and 3 MMW	23.3%
Between 3 (more than 3) and 4 MMW	6.7%
More than 4 MMW	5.6%
*Origin*	
From a rural area	6.9%
From a village	32.7%
From a city	60.4%

**Fig. 1:**
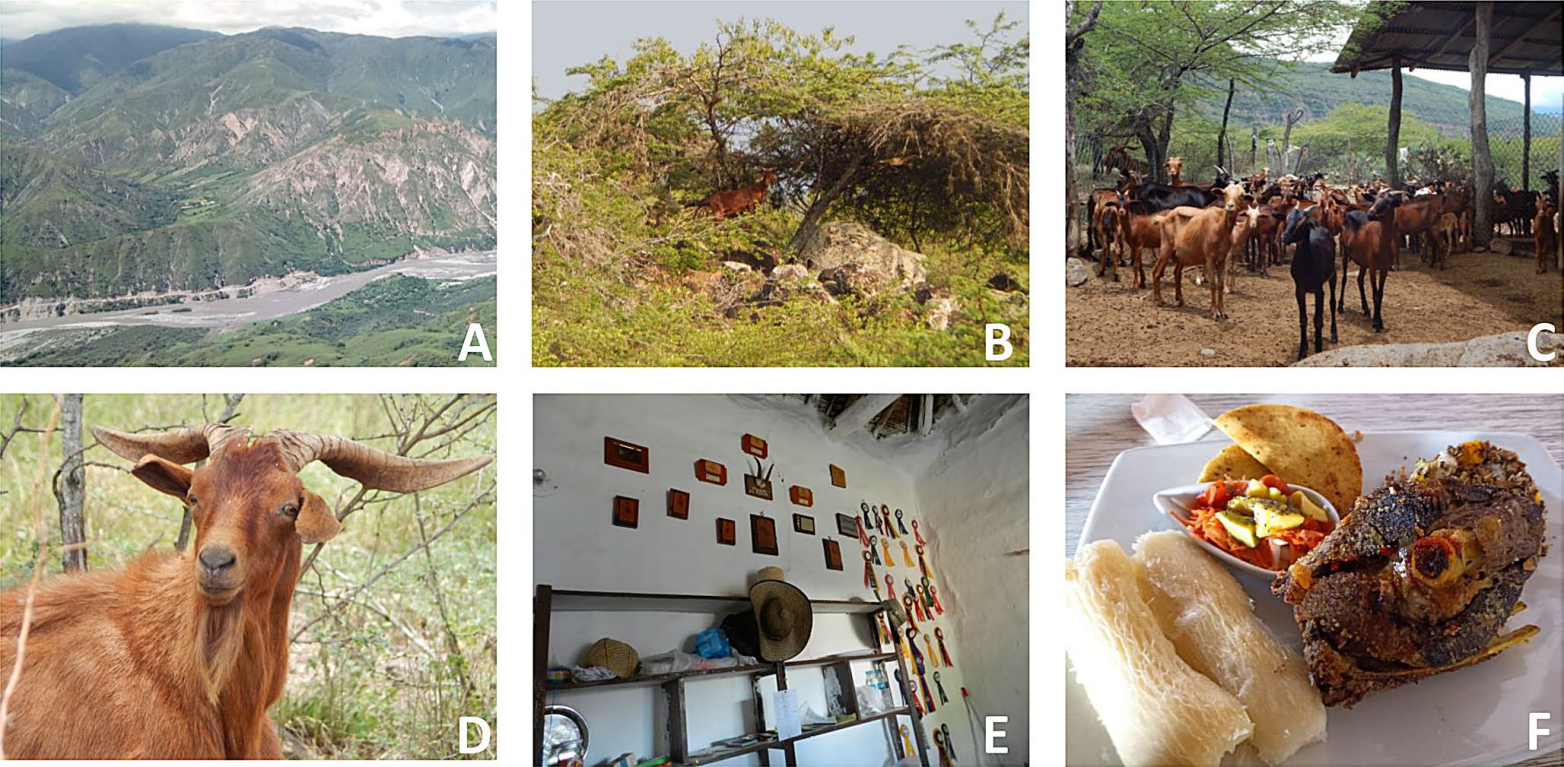
Example of cards used in the choice experiment

A total of 16 cards were developed for the choice-test, each representing a hypothetical product. Each card presented three options: option A, option B, or no option, neither A nor B (Fig. 1). The hypothetical product profiles were constructed using an orthogonal design to achieve a correct balance of attributes, as recommended by Street et al. (2005). The attributes considered in the test were meat type, carcass piece, production system, origin and price (Table 2), with the objective of analyzing the effect that these attributes and their respective levels exert on consumers’ utilities. In addition to goat meat, three other categories of sheep meat were included in the study: wool adult sheep, lamb and hair adult sheep. These categories were included because they are often used as substitutes for goat meat when goat meat is not available. Regarding the origin of the meat, three departments were selected where there is a long-standing tradition of goat and sheep meat production and consumption. The regions of Santander, La Guajira and Boyacá were selected for study. In terms of production systems, the two predominant systems in the country were employed: free-range and feedlot. In relation to the commercial cuts, a list of the meat pieces available in the butcher’s shops of the region was utilized. Finally, different reference prices per pound of fresh meat were included. As the choice process is dynamic, with the combinations of attribute levels changing in each choice set to form the hypothetical products submitted to choose, the model is modelled using MacFadden’s conditional logistic regression. The root likelihood index (RLH) was employed as a metric for evaluating the model’s adequacy (Paetz et al. 2019). The t-ratio was utilized to assess the significance of the attributes (El-Bany et al. 2014). SMRT and SPSS software were utilized to conduct data processing and statistical analysis during this phase of the study.

**Table 2:** Attributes and levels of attributes included in the consumer choice-test

Attributes	Attributes levels	Description
Meat type	1 = Wool Sheep	1. Meat from adult sheep
	2 = Lamb	2. Meat from lambs
	3 = Hair Sheep	3. Meat from hair sheep
	4 = Goat	4. Meat of goats
Origin	1 = Santander	Originally from Santander (north-eastern part of the country).
	2 = Boyacá	Origin to Boyacá (central-eastern part of the country)
	3 = La Guajira	Origin to La Guajira (in the northern region of the country)
	4 = No specific	No specific region
Production system	1 = Stabled	Meat from animals reared in confinement
	2 = Free-range	Meat from free-range animals
Carcass cuts	1 = Cutlet	Cutlet
	2 = Ribs	Ribs
	3 = Leg	Leg
	4 = Arm	Arm
Price per pound of fresh meat	2	Two dollars a pound of meat
	3	Three dollars a pound of meat
	4	Four dollars a pound of meat
	5	Five dollars a pound of meat

### Study 2: farmers

In-depth interviews were conducted with 14 livestock farmers located in the municipalities that make up the *Chicamocha* Canyon, where goat rearing is a deeply rooted activity. Snowball sampling was used for the enrolment of participants, as this technique has been recognized as useful for accessing hard-to-reach populations (Faugier and Sargeant 1997). The first interviews were conducted with the three goat farmers with the largest herds in the region (> 300 goats); subsequently, these farmers helped us get in touch with other colleagues willing to participate in the study. The inclusion criteria for the study were that participants identified themselves as goat farmers, owned their own herd (None of the respondents had fewer than 100 goats), and were actively involved in the goat trade. The interviews were conducted using a script previously designed in accordance with the objectives of the study. Four key topics guided the interviews with the producers: (i) production systems and goat feeding practices, (ii) distinctive aspects of the production system in the *Chicamocha* Canyon and the meat produced, (iii) marketing of the meat, and (iv) the history of goat farming in the region. These topics were selected to explore, from the farmers’ perspective, what makes the meat they produce unique. Among the 14 interviewees, nine were men and five were women, with ages ranging from 40 to 80 years. The socio-productive characteristics of the participating farmers are presented in Table 3.

**Table 3:** Socio-productive characteristics of the participants in the study 2

	Percentage
Gender	
Male	64.3%
Female	35.7%
*Age range*	
Between 40 and 50 years old	42.9%
Between 51 and 60 years old	21.4%
Over 60 years old	35.7%
*Study level*	
Basic/ primary	42.9%
Secondary/ technical	42.9%
University	14.2%
*Head count of goats*	
Under 50 goats	21.4%
Between 50 and 100 goats	57.2%
Over 100 goats	21.4%

Due to the homogeneity of the sample in terms of farm characteristics (e.g., small to medium herd size, extensive production systems) and production dynamics (i.e., meat production for local markets), thematic saturation was reached relatively quickly—around interview 12—when no new relevant themes or variations emerged. This criterion guided the decision to finalize the sample at 14 interviews, which was considered sufficient to capture the perspectives of the target population. The interviews were conducted in situ and audio-recorded with prior informed consent. No personal questions were asked beyond those directly related to goat production. The recordings, totaling 6.5 h, were processed using NVivo 12.0 software and analyzed directly from the audio format without full transcription. A thematic analysis approach was applied: analytical dimensions were defined in advance—including key topics, production risks, animal health and disease control, and general aspects of the farm and producer—and coding was performed by assigning relevant audio fragments to dimensions, nodes (categories), and sub-nodes (subcategories), which were created iteratively as the analysis progressed. Each coded section was annotated using the software’s comment function, and transcribed excerpts were used verbatim to support the results.

## Results and discussion

**Fig. 2:**
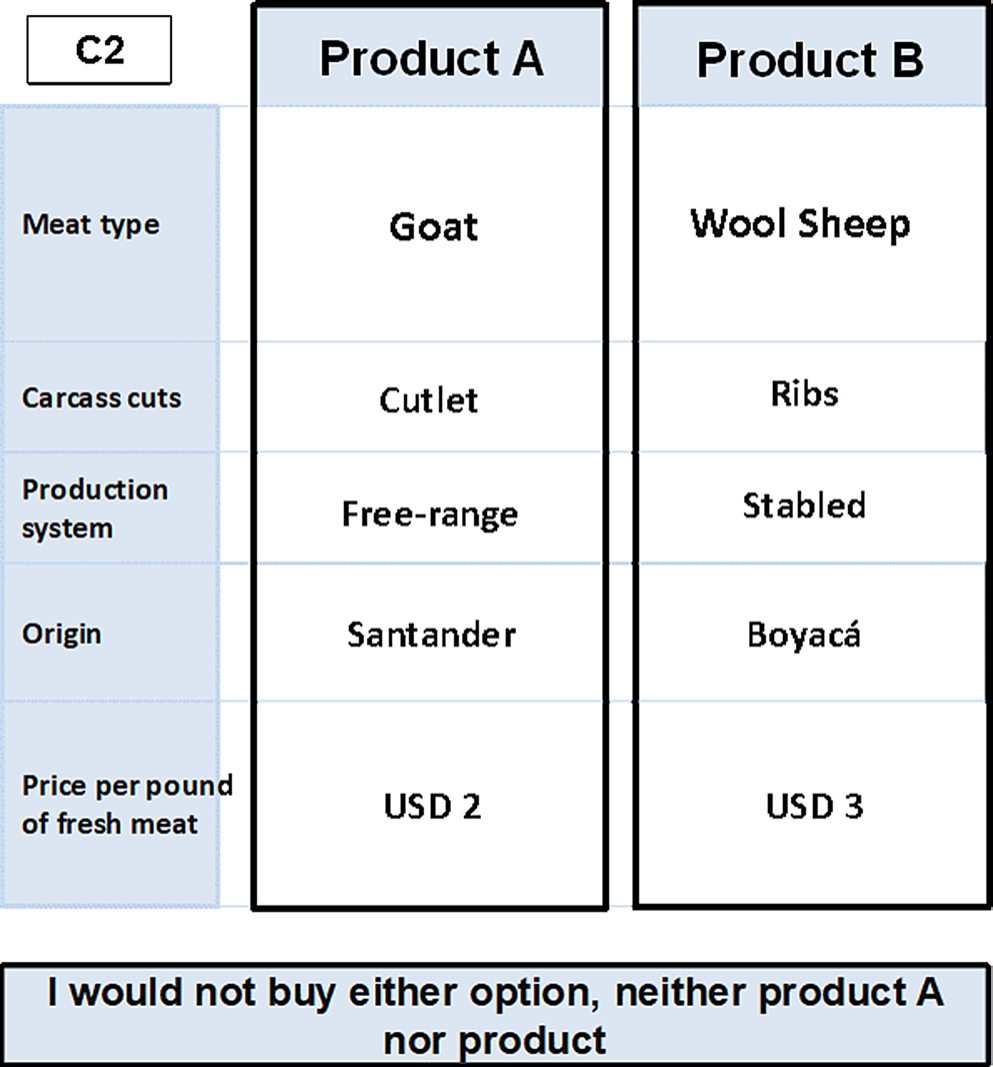
The images presented in this study represent the two extremes of the Santander goat meat supply chain. (**A**) Landscape of the *Chicamocha* Canyon; (**B**) Goats have adapted to the consumption of local fodder and extreme geographical conditions; (**C**) The goat farms in our study are located along the *Chicamocha* Canyon; (**D**) The predominant breed is the *Criollo Santandereana*; (**E**) Farmers in this region actively participate in fairs and competitions of the natve breed; and (**F**) In the culinary tradition of the region, goat meat is regarded as a typical dish of Santander. However, it is also in high demand in traditional restaurants in cities such as Barranquilla, Valledupar, Medellin and Bogotá

In Colombia, the total per capita consumption of the main animal proteins is 76 kg, with chicken meat accounting for 47%, beef for 23%, pork for 17%, fish for 12% and goat meat for 0.3% (FAOSTAT 2024). As in other countries in the Americas, goat meat consumption in Colombia is limited and is predominantly associated with certain Indigenous communities or with arid and semi-arid regions, where it holds strong cultural and culinary significance tied to local traditions and practices (Fig. 2). These dishes cooked with goat meat have become popular in recent years in large cities as part of a rediscovery of traditional cuisine (Estévez-Moreno and Miranda-de la Lama 2022). In this context, our study is a pioneering effort in the region, exploring the perceptions of consumers and producers of goat meat.

### Study 1: consumers

With respect to the frequency of consumption of sheep or goat meat, the results of the study indicate that 56.9% of participants consume it once a year, 34.3% consume it once a month, and the remaining 8.8% have higher consumption frequencies. Table 4 presents the results of the model. The sign of the effect indicates the direction and magnitude of the change in consumer utility because of a one-unit increase in the variable. The t-ratio represents the t-value associated with the significance of each effect. The RLH value of 0.349, the Log-likelihood= -1430.23458 with a Chi-square = 127.75626 for the LR (Likelihood Ratio) test indicates a satisfactory fit of the model. Upon examination of the effects, it becomes evident that price exerts a negative and statistically significant influence on consumers’ profits, a finding that aligns with the tenets of economic theory. Consequently, as the price of products increases, consumers’ profits decrease. Conversely, the negative sign on the variable “None” indicates that consumers derive a higher utility from selecting any option than from selecting none. The results indicate that the selected attributes and levels were perceived by consumers as relevant and worthy of attention.

**Table 4:** Results of the choice test

Attributes levels	Effect	t Ratio
Meat type		
Lamb	0.124	1.911*
Hair Sheep	-0.205	-3.013***
Goat	0.133	2.067*
Wool Sheep	-0.052	-0.792
Carcass cuts		
Leg	-0.076	-1.138
Arm	-0.199	-2.965**
Cutlet	0.130	2.007*
Ribs	0.145	2.232*
Production system		
Free-range	0.045	1.401
Stabled	-0.045	-1.401
Origin		
Santander	0.344	5.386***
La Guajira	-0.312	-4.564**
Boyacá	0.097	1.482
No specific	-0.129	-1.961*
Price	-0.029	-2.503**
None	-0.381	-6.033*

Note: *Significance at 0.05, **Significance at 0.010, *** Significance at 0.001

Our results indicated that within the meat type, lamb and goat had a positive and significant effect on consumer profits, while hair sheep had a negative and significant effect. Therefore, the results indicate that within the types of meat subjected to evaluation, consumers tend to prefer goat meat the most, followed by lamb, while adult hair sheep meat is the least preferred. These results are significant, as there is currently a growing trend in Colombia to import sheep of the Santa Inés, Katahdin, Dorper and Pelibuey breeds to improve local sheep breeds (Aguayo-Ulloa et al. 2022). However, our results demonstrate that consumers exhibit a strong preference for goat meat over sheep meat options. This preference may be attributed to the cultural and gastronomic recognition of goat meat within the region (Serrano-Mejia 2012; Vargas-Bayona et al. 2016a, b). In other words, greater cultural connection may lead to greater preference. Indeed, Semuguruka et al. (2020) found in a study conducted in Tanzania in a highly goat-producing region that goat meat was preferred over beef, pork, chicken and sheep meat. In contrast, Green et al. (2005) reported that compared to other meats such as beef, pork and sheep, consumers held negative attitudes towards its consumption. These results suggest that goat meat has generalized consumption constraints, but that these are overlooked when there is a clear cultural link. Indeed, a consumer study conducted in Kenya by Juma et al. (2010) found that goat meat is perceived as a luxury good, with demand increasing as household income rises.

Regarding the preferred carcass cut, the results indicated that chops and ribs have a positive and significant impact on consumer profits. Conversely, the arm is the part of the carcass least preferred by consumers, with a negative and significant effect on profits. In sheep, other research has also indicated a clear consumer preference for ribs over other parts of the carcass (Shaobo et al. 2022). However, Semuguruka et al. (2020) reported that in Tanzania, the most preferred part of the carcass by consumers was the leg. Although food preparations vary according to culture, which may condition the types of pieces demanded, in sheep, ribs have been reported as the most preferred by consumers for roasting. This may partially explain the results of this study (Shaobo et al. 2022). According to Sepúlveda and Mancilla (2022), among goat meat preparations, roast meat is among the most offered in the region where the study was conducted. Although the free-range production system has a positive effect on consumer profits, no significant effects were found. Consequently, it is somewhat surprising that consumers do not fully recognize free-range farming as a differentiating element, or at least that it does not have a significant effect on consumers’ profits. This may be attributed to the fact that free-range farming is a prevalent practice among farmers in the region where the study was conducted (Vargas-Bayona et al. 2019). It is important to note that, in addition to considerations related to the manner in which animals are reared, extensive livestock production systems may also have a differentiating effect via ecosystem services (Lecegui et al. 2023).

About the origin of the meat, while Santander has a positive and significant effect on consumers’ profits, the *“La Guajira”* and non-specific origins have a negative or significant effect. Therefore, the differentiating role that the origin of Santander goat meat can play in the eyes of consumers is highlighted. It should be borne in mind that the consumers who took part in the study come from the same area of origin as the goat meat under evaluation. Furthermore, this region is renowned for its culinary traditions in relation to goat meat at the national level. Consequently, the differentiating effect of origin may account for consumers’ preference for local products (Lecegui et al. 2023). The accompanying Santander origin effect of goat meat on consumers’ utilities enables the development of a PGI in the region, since, at least from the point of view of local consumers, it enjoys recognition. In this context, an investigation into the potential for the development of a PGI for *Chicamocha* goat meat from the perspective of producers is consistent with the findings of the consumer study.

### Study 2: farmers

In order to provide a more comprehensive overview of the consumer perspectives presented in the previous section, this study also investigated the views of goat farmers in the *Chicamocha* Canyon region. Through in-depth interviews, we explored how producers perceive the distinctive attributes of their product, as well as the cultural, environmental, and economic dimensions of goat farming. These insights offer a more nuanced understanding of the local production system and contribute to the ongoing discourse on the potential valorisation of goat meat through mechanisms such as Protected Geographical Indications (PGIs).

#### Perceptions about meat flavor and feeding practices

One of the questions posed to producers was to identify the aspects that differentiate the goat meat they produce. Five of the interviewees agreed that flavor is one of the main attributes that distinguishes the goat meat produced in the study zone. This was expressed through statements such as: “*Chicamocha* goat is different in taste” and “our goat meat tastes better, the other has no flavor.” One interviewee attributed this distinctive flavor to the goats’ consumption of native vegetation. These perceptions align with narratives surrounding PGIs (Protected Geographical Indications) in Europe, where distinctive organoleptic qualities are frequently associated with pasture-based feeding systems (e.g., Ribeiro et al. 2016; Rodrigues and Teixeira 2009). Unlike intensive systems, goats in extensive systems tend to consume a wide variety of wild plants, as their diet is less controlled (Ndona et al. 2024). In the *Chicamocha* Canyon, endemic species such as *Cavanillesia chicamochae* are consumed, and producers emphasize the role of oregano in the flavor. The differential flavors of goat meat are often attributed by farmers largely to the species they eat, especially oregano. One interviewee noted that goats consume a significant amount of oregano, which contributes to the distinctive taste of goat meat produced in the area. However, Valencia-Duarte et al. (2012) found that oregano is one of the least consumed species due to its low palatability. This contradiction between local perceptions and ecological data highlights the need for further applied research. It also illustrates how local knowledge constructs narratives of product identity that may not fully align with scientific findings.

#### Breed identity and environmental adaptation

Producers also identified the Criolla Santandereana breed as a key factor in meat quality. Due to geographic isolation and centuries of adaptation since colonial times, these goats are considered particularly well-suited to the topography and arid conditions of the *Chicamocha* Canyon. One interviewee stated, “the goats graze along the *Chicamocha*, on the cliffs—that is their terrain,” suggesting strong territorial adaptation to a biocultural landscape. A regional study found that the Criolla breed was predominant on 50.6% of farms, while 23.4% had mostly crossbred goats (Vargas-Bayona et al. 2019). The preference for Criollo and crossbred goats is related to their resilience and ease of management under harsh conditions. Scientific literature supports the influence of breed and feeding system on meat’s sensory attributes (Ripoll et al. 2019; Vega-Galán et al. 2021), although some studies, such as Xazela et al. (2011), suggest that genotype alone may not be significant. These considerations are particularly relevant in the context of a potential PGI, as breed conservation and valorization are key elements in product identity. In 2017, Colombia’s Ministry of Agriculture and Rural Development officially recognized the *Criolla Santandereana* breed as national genetic heritage, reinforcing its symbolic and productive value in the region and gradually on a national scale.

#### Slaughter age and market limitations

Most producers indicated that their main focus is meat production, with limited use of milk, which is primarily used to prepare a local alcoholic beverage known as sabajón (goat milk, aguardiente, sugar, cinnamon, and egg). All interviewees reported that male goats are typically slaughtered at a live weight of between 25 and 30 kg, at an age of five to eight months. These findings are consistent with those of Duarte and Joya (2018) and partially with Vargas-Bayona et al. (2019), who reported a slightly older slaughter age. This early slaughter age may be influenced by forage limitations and local preferences for more intense flavors. In contrast, milder flavors from animals slaughtered at 30 to 60 days old are preferred in Mediterranean Europe (Sánudo 2008). These local preferences may explain both the persistence of traditional practices and the difficulty of entering new segments of the national market (Sepúlveda and Mancilla 2022). Producers also noted that slaughter and sales are primarily conducted through traditional butcher shops. This informality has also been reported in other Latin American countries with traditional goat and sheep meat consumption, such as Mexico (Pulido et al. 2019). These localized and informal distribution chains often represent a significant barrier to market diversification and PGI reach (de Araújo et al. 2022).

#### Biocultural heritage and intergenerational knowledge

There was unanimity among interviewees in stating that goat farming in the region is deeply rooted in family tradition. One producer mentioned that her family has raised goats since 1880 and that this practice has been passed down through at least four generations. Another interviewee indicated that her husband’s grandfather, now 85 years old, has been involved in goat farming since childhood. These testimonies reinforce the view of goat farming as a culturally embedded activity in the *Chicamocha* Canyon. Historical records suggest that goats were introduced to the region around 1543 by Spanish colonizers, mainly from the Canary Islands. The goats were primarily sourced from the Canary Islands, where the *Majorera*,* Tinerfeña*, and *Palmera* breeds were prevalent (Capote and Fresno 2016). The persistence of traditional production methods and the intergenerational transmission of knowledge among goat farmers consolidate the notion that goat farming in the region is not only an economic activity but also a form of intangible cultural heritage (Duarte and Joya 2018; Vargas-Bayona et al. 2016a, b). This perspective supports efforts to recognize and protect these local practices through quality schemes such as PGIs.

### Producers and consumers’ views on the feasibility of a PGI

In consideration of the results obtained, the findings derived from studies conducted with consumers and producers reveal a series of complementary perspectives that support the viability of a Protected Geographical Indication (PGI) for goat meat from the *Chicamocha* Canyon. The findings reveal a pronounced consumer preference for goat meat over other meats, particularly when associated with the Santander region, underscoring the symbolic and gastronomic significance of local production. This regional predilection indicates that a PGI associated with origin could enhance consumer confidence and market value (Vandecandelaere et al. 2010). Producers emphasise the use of the local *Criolla Santandereana* breed, traditional feeding practices based on endemic vegetation, and knowledge passed down from generation to generation. This reinforces the uniqueness and cultural roots of production systems. These attributes correspond with the fundamental pillars of PGI schemes, which require a strong relationship between the characteristics of the product, its geographical origin, and traditional practices (Fernández-Sánchez et al. 2024). The apparent disconnection between scientific evaluations (e.g., oregano consumption) and local narratives further exemplifies the influence of cultural identity on the perception of quality, emphasising the necessity to incorporate both scientific and sociocultural dimensions into the design of a PGI. Taken together, these findings suggest that there is potential for the recognition of *Chicamocha* goat meat as a geographically and culturally differentiated product, with the possibility of its valorisation through a PGI scheme.

## Conclusions

The findings of this study indicate the potential for the establishment of a Protected Geographical Indication (PGI) for goat meat in Colombia, particularly in the region of Santander. On the consumer side, the results indicated a distinct predilection for goat meat over sheep meat, with locally produced meat from Santander receiving the highest valuation. This preference is closely tied to cultural familiarity and regional identity, highlighting the importance of origin in consumer choices. From the producer’s standpoint, farmers highlighted the unique characteristics of their product, particularly its flavour, which is attributed to the native vegetation of the *Chicamocha* Canyon, the implementation of extensive grazing systems, and the Criolla Santandereana breed, which is well-adapted to the region’s arid conditions. The findings, when considered collectively, lend support to the existence of a strong link between product characteristics, production practices, and geographic origin – the three key pillars of a PGI. However, the low frequency of goat meat consumption (around 57% reported once per year) signals an important challenge for market development. While a PGI could potentially enhance product visibility and protect local traditions, its success would require complementary efforts to promote cultural appreciation, increase consumer awareness, and develop marketing strategies tailored to both local and broader markets.

## Data Availability

Data can be made available by sending a request email to corresponding authors.
